# Supplementary material for: What makes a city breastfeeding friendly? A qualitative analysis of interviews with breastfeeding women from Europe and Asia
Source: PLoS One. 2025 Jan 13;20(1):e0317374. doi: 10.1371/journal.pone.0317374 (PMC11729961; doi:10.1371/journal.pone.0317374)
Supplement: Attachment — Submitted filename: response to reviewers.pdf [file pone.0317374.s002.pdf]

18 Sep 2024

Dear Editor,

Editor and Reviewers' Comments and Authors' Response

Thank you for the feedback and comments on our submitted manuscript. We appreciate the time and effort taken to improve our manuscript.

Here are our responses (in blue) to the comments, feedback and questions:

1. Ensure that your abstract adheres to our Instructions for Authors' formatting guidelines:

We have checked that our abstract adheres to the instructions as per https://journals.plos.org/plosone/s/submission-guidelines.#loc-abstract

2. Kindly include the requested supplementary file:

We are unable to include the requested supplementary file because our data comprises of transcripts of the interviews. The transcripts may potentially identify the participants or institutions/hospitals/organizations even if all identifying details are removed. Therefore, sharing this openly would breech confidentiality. We have revised our statement on data sharing to: "Data cannot be shared publicly because of confidentiality and restrictions from research ethics committees as the data in this paper comprises confident transcripts of interviews. De-identified data may be made available upon reasonable request from the corresponding author."

3. There is a concern about the use of participants' names in the text. Please clarify whether the consent form explicitly allowed for the publication of their names in the study. Ensuring that participants were fully informed about this aspect of the publication process is crucial for maintaining ethical standards:

We would like to clarify that the names in the manuscript are pseudonyms. We have added this in the text to make this clear (Lines 196-197). Participants were fully aware that we will use pseudonyms when quoting them in any publications and consented to this in the consent form. The identities of all participants are kept confidential according to ethical standards.

4. Clarification is needed on how interview participants were selected from the larger pool of individuals who filled out the online forms. Please provide details on the criteria or process used to choose these participants:

Additional information is added to the methods section to indicate how we selected the participants form the larger pool (Lines 160-167). In general, it was based on our predetermined criteria as well as availability of the participants. The interviews stopped when data saturation was reached.

"Selection of participants for interview:

All the women who completed the online and screening form, and met the inclusion criteria, were contacted for interviews. Initially, the women were contacted based on the timing of receiving the forms. They were contacted within one week of receiving the screening form, and if they responded, interviews scheduled within two weeks. If a participant did not respond to our contact for an interview appointment, the next person with similar characteristics was selected.

As the number of participants grew, preference was then given to those with characteristics not yet met in the earlier participants. This continued until information saturation was met."

5. While the paper mentions obtaining institutional ethical approval, it would be beneficial to specify which ethical guidelines or frameworks were followed (e.g., Declaration of Helsinki, Belmont Report) to enhance the study's ethical transparency:

We have edited it to specify that the study followed the framework of Declaration of Helsinki (Lines 111-116).

"The study was conducted following the principles of the Declaration of Helsinki and received approval from Joint Penang Independent Ethics Committee (No. JPEC 21- 0011, 4 July 2022), as well as declared as review exempt from UCD Human Research Ethics Committee (No: LS-E-22-139-Tan-McAuliffe, 14 July 2022). Electronic written consent was obtained before the commencement of the interviews, which included consent for the recording of the interviews and use of pseudonyms when quoting them"

6. Response to Reviewers' Specific Comments:

Reviewer 1		
Comment	Authors' Response	Reference in the manuscript
The manuscript is well-written overall. However, please review the attached documents and address all the comments and suggestions provided. This will help enhance the quality and clarity of your paper	Thank you.<br>Please see the attached file for responses to the comments found in the document.<br><br>Each of the comments are also addressed below:	
Abstract:<br>The term "warm chain of support" is well-defined, which helps set the context. However, the definition is quite dense and might benefit from being broken down into simpler components or rephrased to ensure it's easily understood by a broad audience.	Thank you. We are unsure about this comment if it's in general or specific to the abstract. We note the other comment on expanding the definition in the background. We have taken that suggestion in the background but have decided to leave this definition as it is in the abstract due to word limit.	
The methods section is concise and describes the sampling technique, participant demographics, and analytical framework used. However, the explanation of "Purposive sampling" could be simplified or elaborated on	Thank you.<br><br>We have replaced the term purposive sampling in the abstract to "A set of selection criteria was applied to...."	Line 22

slightly to ensure all readers understand its significance.		
Introduction:<br>**Explicitly Address the Research Gap:** While you allude to the importance of an enabling environment, directly stating the lack of standardized indicators for breastfeeding-friendly cities would significantly enhance the introduction's impact. Currently, the text implicitly assumes that readers understand this gap in the literature. Consider adding a sentence like, "Despite the recognized need for supportive environments, there is currently a lack of consensus on what constitutes a breastfeeding-friendly city, hindering the development of effective interventions and policies." This addition would clearly articulate the need for your research and its potential contribution to addressing this gap.<br><br>**Seamlessly Introduce the Study's Purpose:** While the abstract states the study's objective, briefly mentioning the purpose within the introduction would create a smoother transition to the subsequent sections and provide readers with a clearer understanding of the research direction. For instance, you could incorporate a sentence like, "This study aims to explore the perspectives of breastfeeding women in Ireland and Malaysia to understand the key elements of an ideal breastfeeding-	Thank you. We have added the suggested sentence:<br>"Despite the recognized need for supportive environments, there is currently a lack of consensus on what constitutes a breastfeeding-friendly city, hindering the development of effective interventions and policies."<br><br>The subsequent sentence was modified so that the thought flows better.<br><br>With regards to the aims and objectives, we agree that it wasn't clear, we have taken your suggestion and changed it to:<br>"This study aims to explore the perspectives of breastfeeding women from two continents – Europe and Asia, to understand the key elements of an ideal breastfeeding-friendly city"<br><br>In addition, to highlight the study's purpose, we moved it to a new paragraph.	Lines 85-88<br><br>Lines 98-99<br><br>Line 98

friendly city." This addition would link the broader context of breastfeeding support to your specific research aims.		
The warm chain concept is crucial to the study, yet the explanation is slightly dense. Consider adding a brief, clear definition or example of how the warm chain operates in practice, which could help readers unfamiliar with the concept to better grasp its significance.	We added a sentence to make the definition clearer: "The warm chain is akin to the cold chain in vaccine delivery whereby a break within the chain would compromise the outcome, in this case, the duration of breastfeeding."<br>We also drew attention to the readers to Figure 1 to visualize what it means. "See Fig 1 for a diagrammatic representation of the warm chain."	Line 59-61<br><br>Line 62-63
The shift towards Public-Patient Involvement is noted, but the importance of this shift could be emphasized more. A brief statement on how incorporating women's perspectives directly influences the effectiveness of breastfeeding support programs could strengthen the rationale for your study.	Thank you for the suggestion. We have added these to strengthen our rationale:<br>"Policies and interventions developed without input from end-users may fail to gain widespread adoption, as they may not adequately address their needs. Similarly, a breastfeeding-friendly city must be designed with the active participation of breastfeeding women."	Lines 94-96
Clarify the difference between "perceived" and "perceive."	We used the term 'perceived' as in the past tense of perceive. It is in the past tense because the perception is based on past experiences.<br><br>The sentence has now been replaced by Line 98-99, which does not have the word "perceived".	
While the you have mentioned obtaining institutional ethical approval, it does not specify which ethical guidelines were followed. Including this information would enhance the ethical transparency of the study.	The manuscript had the details of the ethical approval on a separate page (not part of the main manuscript).<br>We now added details on the ethical approval in the main manuscript:<br>"The study was conducted following the principles of the Declaration of Helsinki and received approval from Joint Penang Independent Ethics Committee (No. JPEC 21- 0011, 4 July 2022), as well as declared as review exempt from UCD Human Research Ethics Committee (No: LS-E-22-139-	Lines 111-116

	Tan-McAuliffe, 14 July 2022). Electronic written consent was obtained before the commencement of the interviews, which included consent for the recording of the interviews and use of pseudonyms when quoting them”	
Although you’ve described the use of purposive sampling and personal contacts to recruit participants. However, there is no discussion of potential biases that might arise from this method, such as selection bias or the influence of the researchers’ networks on the sample diversity. Addressing these concerns would add rigor to the sampling methodology.	Thank you for highlighting this. To note, the researchers’ networks are rather diverse because of the experience of the main researchers in the field of breastfeeding within each country (Ireland and Malaysia)<br><br>We have added to our discussion on limitation the potential selection bias – the women were recruited from among those who are breastfeeding and are likely advocates for breastfeeding because the belong to a similar group: “In addition, given that women were recruited from two large breastfeeding advocate groups, there is a potential for over-representation of like-minded viewpoints.”	Lines 625-627
The interviews:<br>Single interviewer conducted all the interviews. While this ensures consistency, it could also introduce interviewer bias.	Interviewer bias is unlikely to impact or influence the findings of our study. The study explored both the positive and negative aspects of breastfeeding support as experienced by the women. The structured questions also ensured that both sides of the story are being heard.	
The explaination of the coding process and mentions that the lead investigators generated and reviewed the themes. However, it does not address how potential biases from the researchers’ backgrounds and experiences were managed during the analysis. Discussing any steps taken to ensure objectivity, such as peer debriefing or involving an external auditor, would enhance the credibility of the findings.	The researchers’ background and experiences add to the reflexivity of the process. The analyses were completed from our personal lenses, which is the principle of reflexive thematic analysis by Braun & Clarke. We did not include external auditors. However, the themes were presented to the other authors for discussion and all authors, not only the investigators who developed the initial themes, agreed to the findings.<br><br>We added the following to make it clear: “The themes were presented to the remaining authors (JJH, AOS and FMM) to ensure objectivity and address any bias that may have occurred”.	Lines 207-208

Results:<br>The statement that "many of the women would have had their babies born during the full lock-down period" is speculative. Consider providing specific data or revising the language to clarify whether this was confirmed for the participants or is an assumption.	This was a fact confirmed by the participants and not an assumption.<br>We have revised the sentence to:<br>“During this period, both countries were still in partial lockdown due to the COVID-19 pandemic and many of the women had their babies born during the full lock-down period of the pandemic.”	Lines 212-213
You have states that 40 women expressed interest, but only 22 were interviewed. It would be beneficial to explain why the other 18 were not included (e.g., did not meet inclusion criteria, declined, or were not needed after saturation was reached).	We reviewed our dataset, and confirmed that it was 42 (not 40) who expressed interest. The 20 women who were not included either because they could not be reached to arrange for interviews or we had reached saturation, and thus not contacted (4 declined, and we didn’t need to contact 16)<br>Therefore, we have revised the sentence to:<br>“We interviewed a total of 22 women from a variety of ethnic/cultural backgrounds living in Malaysia (n=11) and Ireland (n=11). The equal number in the two countries was coincidental. Four women did not respond to our invitation for an interview and 16 were not needed after selection based on the demographic characteristics and subsequent information saturation.”<br><br>We also added a section describing the selection process with we hope help make this clear.<br>“**Selection of participants for interview:**<br>All the women who completed the online and screening form, and met the inclusion criteria, were contacted for interviews. Initially, the women were contacted based on the timing of receiving the forms. They were contacted within one week of receiving the screening form, and if they responded, interviews scheduled within two weeks. If a participant did not respond to our contact for an	Lines 214-218<br><br>Lines 160-167

	interview appointment, the next person with similar characteristics was selected. As the number of participants grew, preference was then given to those with characteristics not yet met in the earlier participants. This continued until information saturation was met.”	
You indicate that an equal number of participants were interviewed from Malaysia and Ireland (n=11 each). Consider discussing whether this equal representation was intentional and if so, why it was important for the study’s objectives. If it was coincidental, acknowledging this might be useful.	It was not intentional.<br>We added a sentence to acknowledge this.<br><br>“The equal number in the two countries was coincidental.”	Line 216
You notes that participants from Ireland were generally older and more educated compared to those from Malaysia. This demographic difference could have influenced the findings. It would be helpful to mention whether these differences were considered in the analysis or interpretation of the results.	We agree that the demographic differences influenced the findings. We did compare the findings between Ireland and Malaysia and these were discussed in the throughout the Discussion. Lines 563-580 specifically discussed the differences in demographic between the 2 countries.	
Table 1: Kindly move this in the last coloumn make the frist coloumn in Ireland followed by Malaysia in all tables.	Thank you. We have taken up your suggestion and edited the table.	Table 1
Table 1:<br><br>In this row, clarify that "Twins" and "Preterm delivery" are not mutually exclusive. Consider adding a footnote or combining these into a single count if some babies had both complications.	There is a footnote to explain this. We prefer not to combine to show the difference.<br><br>We made it clearer that there’s a footnote to Table 1, to explain that one baby may have more than one complication.	
Consider briefly summarizing the overall findings related to	We presented a summary of our overall findings with the subthemes in Table 2.	

this theme before introducing the subthemes. This would provide a clearer foundation for understanding the subsequent details.	We made it clearer that a summary can be found in Table 2:<br>"A summary of the overall findings is presented in Table 2".	Lines 229-230
The mothers expressed disappointment when breastfeeding was secondary during sickness. It would be beneficial to explore why healthcare providers might prioritize formula feeding in these situations and how this approach could be improved.	We did not explore this with our data because it was not the objective of our study. While it is important, we would not like to make any suggestions here because it's not supported by our data (our data not designed to determine this)	
Consider adding a recommendation or suggestion based on the mothers' feedback about the lower priority of breastfeeding in certain cases. This could provide actionable insights for improvement.	Thank you but we do not agree with this. The aim of the study is to present the evidence. It is not our intention to provide suggestions for improvement.	
When quoting Ruby and Olivia, a brief interpretation or reflection on why breastfeeding rooms were not prioritized could enhance the analysis.	We have added a brief interpretation of why that happened.<br><br>"These examples highlighted that the people who provided the breastfeeding rooms had not thought about the usability of the rooms or understood how breastfeeding works"	Lines 327-328
Despite differences between Ireland and Malaysia, the general need for a supportive breastfeeding environment was consistent. However, specific challenges, such as public acceptance and workplace support, differed. For instance, Irish women enjoyed longer maternity leave and breastfeeding breaks compared to Malaysian women, who faced more limited support.	Thank you for this comment. We agree and had addressed this in our discussion in lines 563-580.	
Reviewer 2		

Comment	Authors' Response	Reference in the manuscript
Thank you for requesting me to review the paper "What makes a city breastfeeding friendly? A qualitative analysis of interviews with breastfeeding women from Europe and Asia".<br>General comments<br>This is a great work and well-written. There are various points where participant's names were mentioned. I want to inquire whether the consent form specified that their name should appear in the publication.	Thank you very much for the kind words.<br><br>We would like to clarify that the names in the manuscript are pseudonyms. Participants consented to their quotes published using pseudonyms.<br><br>We have added this to the methods section to make it clear.<br>"To avoid identification, we used pseudonyms when quoting the participants"	Lines 191-192
Lines 143 – 149, when was the demographic characteristics data collected (Dates started and ended)?	The demographic data was collected at the point of consent. The interviews took place on an average 2 weeks after the consent. Therefore, the date of the demographic data collected correspond with the interview dates as stated in the manuscript.<br><br>We added a new section "Selection of participants for interview" to clarify this.	Lines 160-167
Methods: Were the interviews conducted during the period for demographic characteristics data collection? How were the participants for interviews chosen from the many participants who filled in the online forms?	As above regarding the demographic data.<br><br>The participants were selected based on our predetermined criteria and availability of the participants<br><br>We added a section on how the participants were selected for the interview: "Selection of participants for interview"	Lines 160-167
Line 189 – 191 can be transferred to the methods section.	Thank you for the suggestion. After consideration, we decided that these lines are more related to results than methods.	
Selection criteria, line 192 can also be taken to the methods section.	Thank you for the suggestion. After consideration, we decided to keep this line at its current place. We added a section in the methods explaining the selection process: Lines 160-167	

The discussion was generally well done.	Thank you once again.	

Thank you.

Sincerely,

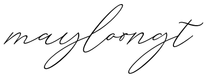

May Loong Tan

Corresponding Author
