# Supplementary material for: What makes a city breastfeeding friendly? A qualitative analysis of interviews with breastfeeding women from Europe and Asia
Source: PLoS One. 2025 Jan 13;20(1):e0317374. doi: 10.1371/journal.pone.0317374 (PMC11729961; doi:10.1371/journal.pone.0317374)
Supplement: Attachment — Submitted filename: PONE-D-24-22876_reviewer .docx [file pone.0317374.s001.docx]

PLOS ONE

What makes a city breastfeeding friendly? A qualitative analysis of interviews with breastfeeding women from Europe and Asia

--Manuscript Draft--

| Manuscript Number: | PONE-D-24-22876 |
| --- | --- |
| Article Type: | Research Article |
| Full Title: | What makes a city breastfeeding friendly? A qualitative analysis of interviews with breastfeeding women from Europe and Asia |
| Short Title: | Breastfeeding friendly city: a qualitative study of women in Europe and Asia |
| Corresponding Author: | May Loong Tan University College Dublin IRELAND |
| Keywords: | breastfeeding; warm chain; support; qualitative; city |
| Abstract: | Background: The warm chain of support is the continuous enabling environment from the mother’s first contact with healthcare professionals during early pregnancy, birth and immediate post-partum period, her transition from healthcare facility to home, through to work and the community at large. A breastfeeding-friendly city should be able to support a breastfeeding journey across the warm chain.  Objective: To determine breastfeeding women’s perspective of an ideal breastfeeding- friendly city.  Methods: Between September 2021 and January 2022, twenty-two women who were breastfeeding or had ever breastfed in the last 5 years from Ireland and Malaysia were interviewed. Purposive sampling was applied to ensure representation of a range of the characteristics known to be associated with breastfeeding success: diverse age groups, birth and breastfeeding experiences, culture and socioeconomical background. One-on-one semi-structured online interviews were conducted by the first author. Data were analysed using Braun and Clarke’s Thematic Analysis framework.  Results: One overarching theme of breastfeeding at the front and centre of the city, with three major themes were developed: 1. mothers feel supported when breastfeeding is prioritised; 2. when breastfeeding is visible in the environment, it becomes normalized; 3. There is a need to have seamless breastfeeding support across the continuum of the warm chain, and at all levels of society.  Conclusions: The findings demonstrated the importance of prioritized, and continuous support throughout the breastfeeding journey. The hopes and aspirations of a breastfeeding-friendly city expressed here would be useful for cities to consider when developing or implementing breastfeeding support programmes as well as guide development of indicators of a breastfeeding-friendly city. |
| Order of Authors: | May Loong Tan |
|  | Elizabeth J O'Sullivan |
|  | Jacqueline J Ho |
|  | Amal Omer-Salim |
|  | Fionnuala M McAuliffe |
| Additional Information: |  |
| Question | Response |
| Financial Disclosure  Enter a financial disclosure statement that describes the sources of funding for the work included in this submission. Review the [submission guidelines](http://journals.plos.org/plosone/s/submission-guidelines#loc-financial-disclosure-statement) for detailed requirements. View published research | The author(s) received no specific funding for this work. |

*Powered by Editorial Manager® and ProduXion Manager® from Aries Systems Corporation*

| articles from [*PLOS ONE*](https://journals.plos.org/plosone/) for specific examples.  This statement is required for submission and will appear in the published article if the submission is accepted. Please make sure it is accurate. |  |
| --- | --- |
| Funded studies  Enter a statement with the following details:   - Initials of the authors who received each award - Grant numbers awarded to each author - The full name of each funder - URL of each funder website - Did the sponsors or funders play any role in the study design, data collection and analysis, decision to publish, or preparation of the manuscript? |  |
| Did you receive funding for this work? |  |
| Competing Interests  Use the instructions below to enter a competing interest statement for this submission. On behalf of all authors, disclose any [competing interests](http://journals.plos.org/plosone/s/competing-interests) that could be perceived to bias this work—acknowledging all financial support and any other relevant financial or non- financial competing interests.  This statement is required for submission and will appear in the published article if the submission is accepted. Please make sure it is accurate and that any funding sources listed in your Funding Information later in the submission form are also declared in your Financial Disclosure statement.  View published research articles from  [*PLOS ONE*](http://journals.plos.org/plosone/) for specific examples. | The authors have declared that no competing interests exist. |

*Powered by Editorial Manager® and ProduXion Manager® from Aries Systems Corporation*

| NO authors have competing interests  Enter: *The authors have declared that no competing interests exist.*  Authors with competing interests  Enter competing interest details beginning with this statement:  *I have read the journal's policy and the authors of this manuscript have the following competing interests: [insert competing interests here]* |  |
| --- | --- |
| * typeset |  |
| Ethics Statement  Enter an ethics statement for this submission. This statement is required if the study involved:   - Human participants - Human specimens or tissue - Vertebrate animals or cephalopods - Vertebrate embryos or tissues - Field research   Write "N/A" if the submission does not require an ethics statement.  General guidance is provided below. Consult the [submission guidelines](http://journals.plos.org/plosone/s/submission-guidelines#loc-guidelines-for-specific-study-types) for detailed instructions. Make sure that all information entered here is included in the Methods section of the manuscript. | The study was conducted following the principles of the Declaration of Helsinki and received approval from Joint Penang Independent Ethics Committee (No. JPEC 21- 0011, 4 July 2022), as well as declared as review exempt from UCD Human Research Ethics Committee (No: LS-E-22-139-Tan-McAuliffe, 14 July 2022). Electronic written consent was obtained before the commencement of the interviews, which included consent for the recording of the interviews. |

*Powered by Editorial Manager® and ProduXion Manager® from Aries Systems Corporation*

| Format for specific study types  Human Subject Research (involving human participants and/or tissue)   - Give the name of the institutional review board or ethics committee that approved the study - Include the approval number and/or a statement indicating approval of this research - Indicate the form of consent obtained (written/oral) or the reason that consent was not obtained (e.g. the data were analyzed anonymously)   Animal Research (involving vertebrate animals, embryos or tissues)   - Provide the name of the Institutional Animal Care and Use Committee (IACUC) or other relevant ethics board that reviewed the study protocol, and indicate whether they approved this research or granted a formal waiver of ethical approval - Include an approval number if one was obtained - If the study involved *non-human primates*, add *additional details* about animal welfare and steps taken to ameliorate suffering - If anesthesia, euthanasia, or any kind of animal sacrifice is part of the study, include briefly which substances and/or methods were applied   Field Research  Include the following details if this study involves the collection of plant, animal, or other materials from a natural setting:   - Field permit number - Name of the institution or relevant body that granted permission |  |
| --- | --- |
|  |  |
| Data Availability  Authors are required to make all data underlying the findings described fully available, without restriction, and from the time of publication. PLOS allows rare exceptions to address legal and ethical concerns. See the [PLOS Data Policy](http://journals.plos.org/plosone/s/data-availability) and [FAQ](http://journals.plos.org/plosone/s/data-availability#loc-faqs-for-data-policy) for detailed information. | No - some restrictions will apply |

*Powered by Editorial Manager® and ProduXion Manager® from Aries Systems Corporation*

| A Data Availability Statement describing where the data can be found is required at submission. Your answers to this question constitute the Data Availability Statement and will be published in the article, if accepted. |  |
| --- | --- |
| Important: Stating ‘data available on request from the author’ is not sufficient. If your data are only available upon request, select ‘No’ for the first question and explain your exceptional situation in the text box. |  |
| Do the authors confirm that all data underlying the findings described in their manuscript are fully available without restriction? |  |
| Describe where the data may be found in full sentences. If you are copying our sample text, replace any instances of XXX with the appropriate details.   - If the data are held or will be held in a public repository, include URLs, accession numbers or DOIs. If this information will only be available after acceptance, indicate this by ticking the box below. For example: *All XXX files are available from the XXX database (accession number(s) XXX, XXX.*). - If the data are all contained within the manuscript and/or Supporting Information files, enter the following: *All relevant data are within the manuscript and its Supporting Information files.* - If neither of these applies but you are able to provide details of access elsewhere, with or without limitations, please do so. For example:   *Data cannot be shared publicly because of [XXX]. Data are available from the XXX Institutional Data Access / Ethics Committee (contact via XXX) for researchers who meet the criteria for access to confidential data.*  *The data underlying the results presented in the study are available from (include the name of the third party* | Data cannot be shared publicly because of confidentiality. De-identified data may be made available upon reasonable request from the corresponding author. |

*Powered by Editorial Manager® and ProduXion Manager® from Aries Systems Corporation*

| *and contact information or URL).*   - This text is appropriate if the data are owned by a third party and authors do not have permission to share the data.   * typeset |  |
| --- | --- |
| Additional data availability information: |  |

*Powered by Editorial Manager® and ProduXion Manager® from Aries Systems Corporation*

Manuscript	[Click here to access/download;Manuscript;Manuscript Final_](https://www2.cloud.editorialmanager.com/pone/download.aspx?id=36550363&guid=fe8ee3c2-662c-4f45-aa1d-a98252c56c88&scheme=1)[<https://www2.cloud.editorialmanager.com/pone/download.aspx?id=36550363&guid=fe8ee3c2-662c-4f45-aa1d-a98252c56c88&scheme=1> What makes a city breastfeeding friendly.docx](https://www2.cloud.editorialmanager.com/pone/download.aspx?id=36550363&guid=fe8ee3c2-662c-4f45-aa1d-a98252c56c88&scheme=1)

1	**What makes a city breastfeeding friendly? A qualitative**

2	**analysis of interviews with breastfeeding women from**

3	**Europe and Asia**

4

5	May Loong Tan ^1,^ ^2*^, Elizabeth J O’Sullivan ^3^, Jacqueline J Ho ^2^, Amal Omer-Salim^4^, Fionnuala M

6 McAuliffe ^1^
7 ^1^ UCD Perinatal Research Centre, School of Medicine, University College Dublin, National Maternity
8 Hospital, Dublin, Ireland
9 ^2^ Department of Paediatrics, RCSI & UCD Malaysia Campus, Penang, Malaysia
10 ^3^ School of Biological, Health and Sports Sciences, Technological University Dublin, Ireland
11 ^4^ WABA: World Alliance for Breastfeeding Action
12 * Corresponding author
13 [may.tan@ucdconnect.ie](mailto:may.tan@ucdconnect.ie), [mltan@rcsiucd.edu.my](mailto:mltan@rcsiucd.edu.my) (MLT)

# 14 Abstract:

15 Background: The warm chain of support is the continuous enabling environment from the mother’s
16 first contact with healthcare professionals during early pregnancy, birth and immediate post-partum
17 period, her transition from healthcare facility to home, through to work and the community at large.
18 A breastfeeding-friendly city should be able to support a breastfeeding journey across the warm chain.
19 Objective: To determine breastfeeding women’s perspective of an ideal breastfeeding-friendly city.
20 Methods: Between September 2021 and January 2022, twenty-two women who were breastfeeding or
21 had ever breastfed in the last 5 years from Ireland and Malaysia were interviewed. Purposive
22 sampling was applied to ensure representation of a range of the characteristics known to be associated
23 with breastfeeding success: diverse age groups, birth and breastfeeding experiences, culture and
24 socioeconomical background. One-on-one semi-structured online interviews were conducted by the
25 first author. Data were analysed using Braun and Clarke’s Thematic Analysis framework.
26 Results: One overarching theme of breastfeeding at the front and centre of the city, with three major
27 themes were developed: 1. mothers feel supported when breastfeeding is prioritised; 2. when
28 breastfeeding is visible in the environment, it becomes normalized; 3. There is a need to have
29 seamless breastfeeding support across the continuum of the warm chain, and at all levels of society.
30 Conclusions: The findings demonstrated the importance of prioritized, and continuous support
31 throughout the breastfeeding journey. The hopes and aspirations of a breastfeeding-friendly city
32 expressed here would be useful for cities to consider when developing or implementing breastfeeding
33 support programmes as well as guide development of indicators of a breastfeeding-friendly city.

34

# 35 Introduction:

36 Breastfeeding is essential for the health and well-being of the newborn and the mother, with short-
37 and long-term benefits for both (1-3). Beyond health, the economic benefits of a community with
38 high breastfeeding rates are also significant (4). The optimal duration of breastfeeding is exclusive for
39 the first 6 months and continued until 2 years and beyond (5). Despite these, not every mother who
40 has the intention to breastfeed will succeed (6, 7). Breastfeeding is not always easy, and multiple
41 factors contribute to the early cessation of breastfeeding. Reasons such as latching difficulties, pain,
42 perceived milk insufficiency, and work were universally reported by mothers, including those in
43 Ireland and Malaysia, as reasons to stop breastfeeding before the recommended duration of at least 2
44 years (8-10). There are also evidence showing how non-breastfeeding-friendly practices, such as early
45 supplementation of formula in the hospital, can shorten the duration of breastfeeding despite good
46 maternal intentions (11). Apart from these, factors such as wellbeing, self-efficacy, adequate sleep,
47 supportive partner, and healthcare professionals, also contributes to breastfeeding outcomes (2, 12-
48 14). Seeing that internal and external factors contribute to success of breastfeeding, it is imperative
49 that an enabling environment throughout the breastfeeding journey is in place
50 for each woman and her family.
51 An enabling environment for breastfeeding starts from the woman’s first contact with healthcare
52 professionals during early pregnancy. It continues at birth and immediate post-partum period, and
53 subsequently her transition from the healthcare facility to home, work, and the community. The
54 community includes public spaces such as parks, public transport as well as other ‘open’ places a
55 breastfeeding woman might visit such as markets, shops, restaurants, and places of worship. The
56 enabling environment could also precede these present-day environments, such as early exposure to
57 breastfeeding as a child and education on breastfeeding in school. The continuation and connectivity
58 of these diverse supports through the duration of breastfeeding have been referred to as the warm
59 chain of support for breastfeeding (15). Within the warm chain are ‘actors’, who are people that play a
60 role in extending support to the mother-infant dyad at a specific point of time (Fig 1).
61 **Fig 1: Diagrammatic representation of the warm chain of support for breastfeeding (adapted**
62 **with permission from World Alliance for Breastfeeding Action (WABA)**

## 63 The conceptual models of determinants of breastfeeding

64 One of the earliest conceptual models of determinants of breastfeeding was the ecological breastfeeding
65 model described by Hector *et al*. (16). In this framework, the authors described breastfeeding as being
66 influenced by individual level, group level and society level factors. Individual level factors include
67 intentions, past experience, birth experience, and health. Group level factors relate to the environment
68 within which the mother and baby reside in such as healthcare, community, and workplace. The society
69 level factors referred to cultural norms related to breastfeeding, parenting, and women in general.
70 A more recent model of the determinants of breastfeeding was described by the authors of the Lancet
71 Breastfeeding Series 1 (17). In this framework, three levels of determinants influence a mother’s
72 breastfeeding behaviour – structural, settings, and individual. The structural determinants are the
73 fundamental background influences of social and culture, shaped not only by cultural beliefs but also
74 by marketing of infant formula. The settings described in this model were healthcare, community, and
75 workplace. The individual level relates to the mother-infant dyad as well as their relationship with other
76 people around them. The warm chain concept is a step up from these models, with the emphasis on the
77 connection between all the different components of the determinants of breastfeeding. A breastfeeding-
78 friendly city is an example of how elements of these different models are operationalized
79 simultaneously.
80 In line with a call to scale up breastfeeding support (18), cities around the world have become
81 “Breastfeeding-friendly” or declared themselves to be such (19-21). However, different criteria and
82 definitions have been used for each city and there are no consistent and specific indicators of a
83 breastfeeding-friendly city (22). Our research team set out to develop a novel set of indicators of a
84 breastfeeding-friendly city. Generally, indicators for breastfeeding support programmes or
85 interventions have not consistently included the perspectives of breastfeeding women. This included
86 the early versions of the Baby Friendly Hospital Initiative (23). This is not surprising as Public-
87 Patient-Involvement or co-production of research was not common practice until the last few years
88 (24). As breastfeeding women are ‘users’ of the breastfeeding friendly cities, they should be consulted
89 to find out what matters to them. Therefore, the aim of this study is to understand what women
90 perceived as a breastfeeding-friendly city and environment. The objective of this study is to develop
91 an in-depth understanding of what a breastfeeding friendly city is, from the perspective of
92 breastfeeding women across two continents - Europe and Asia. A list of items obtained from the
93 interviews were used in a Delphi study as part of the development of a new indicator set of as
94 breastfeeding-friendly city (25). This report describes the detailed, inductive analysis of the
95 interviews.

# 96 Methods:

97 To achieve our objective, we conducted one-on-one semi-structured online interviews with a diverse
98 group of women from Ireland in Europe and Malaysia in Asia.
99 Women were eligible to participate if they were over 18 years old and had ever breastfed a baby in the
100 last 5 years. “Ever breastfed” was defined according to WHO Indicators for Assessing Infant and
101 Young Child Feeding as anyone who had provided her own or another baby (i.e., adopted baby or wet
102 nursing) any amount of breastmilk directly from her breasts or as expressed breastmilk (26).
103 Participants were ineligible if they were unable to converse in English or Malay or had no internet
104 access for the interview. All participants gave written consent prior to the interviews. The study
105 obtained institutional ethical approval prior to recruitment.

## 106 The setting:

107 This study was conducted with participants from Ireland and Malaysia. Ireland is a European, high-
108 income country with a low breastfeeding rate (31.1% exclusive breastfeeding at 3 months, 2021) (27)
109 while Malaysia is a Southeast Asian, upper-middle income country with a modestly higher
110 breastfeeding rate (47.1% prevalence of exclusive breastfeeding under 6 months, 2016) (28).
111 Although both countries have a national breastfeeding policy in place, the differences in the cultural
112 and economic background of the two countries could have influenced the practice of breastfeeding.
113 Therefore, including participants from these countries provided a global perspective to the subject as
114 well as an opportunity to compare differences in views.

## 115 Sampling method and sample size:

116 Information about the study was circulated to two online (Facebook) breastfeeding support groups, one
117 in Ireland (posted on 24 September 2021) and the other in Malaysia (posted on 30 August 2021). Prior
118 to posting the information about the study on the groups’ Facebook pages, the administrators of the
119 groups were contacted for approval. Collectively, the two breastfeeding groups had over 120,000
120 members. In the post on Facebook, we asked eligible and interested women to contact the primary
121 investigator. Women who responded to the call to participate were asked to complete a screening
122 questionnaire to identify a sample that was diverse based on the characteristics below (listed in
123 alphabetical order). The characteristics were selected based on known individual level factors
124 associated with breastfeeding (29, 30).
125 1. Age
126 2. Breastfeeding experience (first time, had breastfed more than 1 child)
127 3. Education background (highest educational attainment)
128 4. Employment status (full time employment, stay-at-home mother)
129 5. Ethnic and cultural background
130 6. Had a preterm baby and/or twins
131 7. How they breastfeed (direct breastfeeding, expressed breast milk feeding)
132 8. Weight and height for calculation of body mass index
133 To obtain the perspectives of a wide range of women who differed on characteristics known to be
134 associated with breastfeeding, we used purposive sampling. We reviewed the characteristics of the
135 participants and selected them for the interviews based on the characteristics. The decision for
136 selection of a participant was made by the lead investigator in consultation with the other
137 investigators. As the interviews went on, personal contacts of the investigators were also used to
138 source participants with the predetermined characteristics that were yet represented in the study.
139 The sample size was determined by two factors: ethnographic and information saturation. We
140 recruited participants until all categories of the characteristics of women listed above were
141 represented. The interviews also continued until the point where no new information about what
142 makes a city breastfeeding-friendly was forthcoming.

## 143 Demographic data collection:

144 Women who expressed an interest and consented to be interviewed, completed an online screening
145 form before the interview. The screening form collected demographic data such as age (categorical),
146 ethnic background, education level, employment status, method of breastfeeding (direct, expressed or
147 combination of both), past breastfeeding experience, self-reported weight, self-reported height, and
148 details about their baby (prematurity (less than 37 weeks gestation), twins or admission to neonatal
149 intensive care). The women were not given any renumeration for their participation.

## 150 The interviews:

151 The interviews were conducted by a single interviewer (MLT from Malaysia) *via* a video
152 conferencing platform (Zoom). One participant from Malaysia was interviewed over the phone
153 because she did not know how to use a video conferencing application despite having internet access.
154 This was reported back to the ethics committee at the end of the study.
155 The interviews were conducted either in English or Malay, according to the participants’ preference
156 and were recorded (video and audio) using the record function of the video conferencing platform.
157 MLT is fluent in both languages. The sole telephone interview was recorded using an external audio
158 recorder. The interviews were structured to ask about the supportive or enabling factors for
159 breastfeeding at healthcare facilities, workplaces (if working) and public areas, negative breastfeeding
160 experiences at healthcare facilities, workplaces (if working) and public areas, and expectations of an
161 ideal breastfeeding-friendly city. The interview questions were pre-tested in a separate group of
162 breastfeeding women from Ireland and Malaysia prior to the study (3 Malaysians, 1 Irish). The pre-
163 testing was to ensure that the questions were clear in both the English and Malay languages. After the
164 pre-testing, minor modifications were made to the questions, and additional probes were included. For
165 example, we added questions about transitioning from hospital to home and support during the first 6
166 weeks. See Box 1 for the questions and probes.
167 **Box 1: Questions and probes used in the semi-structured interviews.**

I am doing a study to develop markers/indicators of a breastfeeding friendly city. Because a mother moves through different sectors that exist in a city, I would like to hear your experiences relating to breastfeeding in various places.

Before we start, could you please tell me a bit about yourself and your breastfeeding journey thus far?

Let’s start with your experiences in the hospital setting. What do you recall that was helpful to you? What was not helpful?

What about your experience transitioning from hospital to home? What about support at home in the first 6 weeks?

Next, let’s talk about work. In your work, what was helpful? What was not? What about transitioning from home to work?

We now move to the last setting – public places, these can include shops, restaurants, churches etc. What was helpful? What was not?

Let’s say your city is now declared breastfeeding friendly. What does it look like to you? What do you think we should do to achieve that?

Do you understand what is breastfeeding friendly?

168

## 169 Data management and analysis:

170 All interview recordings were transcribed verbatim in the original language used. All transcriptions
171 were pseudo-anonymised before translations and analysis. Transcriptions of interviews conducted in
172 Malay was translated into English by MLT and were cross-checked by an independent person (a
173 colleague of MLT who is fluent in both languages).
174 We used NVivo 13 to organize and manage the data analysis (31). The qualitative analysis was
175 conducted using reflexive thematic analysis as described by Braun & Clarke (32). After the initial
176 reading of the transcripts, the data were grouped into the pre-specified sectors of a city (hospital,
177 workplace, community, and public spaces). This also helped with the familiarization with the data.
178 Next, data were coded in two phases: first, within each sector and subsequently across the entire
179 dataset irrespective of sector. The coding process used both semantic (mainly for codes within a
180 sector) and latent (mainly for codes across the dataset) codes. MLT conducted the grouping and
181 coding as well generating the initial themes. MLT and EJOS then reviewed the initial themes together
182 and defined them further. Both MLT and EJOS (from Ireland) are active researchers and advocates
183 for breastfeeding and have constant interactions with breastfeeding women in their professional
184 capacities. They had also breastfed their own children.
185 Throughout the process of defining the themes, we used an inductive approach, i.e. observation (what
186 the mother reports), pattern recognition (what mothers report in all sectors) and theory development
187 (final themes). All authors reviewed and agreed on the final themes.

# 188 Results:

189 Interviews were conducted between September 2021 and January 2022 with women from Malaysia
190 and Ireland. During this period, both countries were still in partial lockdown due to the COVID-19
191 pandemic and many of the women would have had their babies born during the full lock-down period
192 of the pandemic. Forty women expressed interest in participating in the study. After selection based
193 on the demographic characteristics and subsequent information saturation, we interviewed a total of
194 22 women from various ethnic/cultural backgrounds living in Malaysia (n=11) and Ireland (n=11).
195 Eighteen interviews were conducted in English and four in Malay language. The interviews lasted an
196 average of 16:24 minutes (range 9:30 to 20:30 minutes). The shorter interviews involved participants
197 who were less forthcoming and needed a lot of prompting. All the predetermined characteristics were
198 present among the participants. The participants from Ireland were generally older with higher
199 education level compared to the Malaysians. Table 1 shows a summary of the characteristics of
200 participants.
201 Table 1: Demographic and characteristics of participants

| Characteristics | Total (n=22) | Ireland (n=11) | Malaysia  (n=11) |
| --- | --- | --- | --- |
| Age |  |  |  |
| Between 21 -30 years old | 7 | 2 | 5 |
| Between 31 – 40 years old | 14 | 9 | 5 |
| Above 40 years old | 1 | 0 | 1 |
| Ethnicity/Cultural background |  |  |  |
| White (Irish) | 9 | 9 | 0 |
| White (Mexican) | 1 | 1 | 0 |
| Asian (Malay) | 4 | 0 | 4 |
| Asian (Chinese) | 5 | 1 | 4 |
| Asian (Indian) | 1 | 0 | 1 |
| Asian (Filipino) | 1 | 0 | 1 |
| Body Mass Index |  |  |  |
| Less than 24.9 kg/m^2^ | 13 | 6 | 7 |
| Between 25 to 29.9 kg/m^2^ | 6 | 3 | 3 |
| Equal or above 30 kg/m^2^ | 3 | 2 | 1 |
| Highest Education attainment |  |  |  |

| Certificate or Diploma | 3 | 0 | 3 |
| --- | --- | --- | --- |
| Bachelor’s Degree | 10 | 3 | 7 |
| Higher degree (Masters or PhD) | 9 | 8 | 1 |
| Employment status |  |  |  |
| Full-time | 14 | 10 | 4 |
| Part-time | 3 | 1 | 2 |
| Self-employed | 1 | 0 | 1 |
| Stay-at-home mother | 4 | 0 | 4 |
| Neonatal complications ^a^ |  |  |  |
| Twins | 2 | 0 | 2 |
| Preterm delivery | 5 | 3 | 2 |
| Baby in NICU | 6 | 3 | 3 |
| Past breastfeeding experience |  |  |  |
| First-time breastfeeding | 6 | 4 | 2 |
| Breastfed 2 children | 8 | 6 | 2 |
| Breastfed 3 or more children | 8 | 1 | 7 |
| Method of breastfeeding |  |  |  |
| Only direct feeding | 9 | 7 | 2 |
| Mixed direct and expressed feeding | 13 | 4 | 9 |
| Only expressed breast milk feeding **^b^** | 1 | 1 | 0 |

202 ^a^ One baby may have more than one complication.
203 ^b^ One of 3 children.

## 204 Overarching theme: Breastfeeding front and centre

205 From the interviews, we developed one overarching theme and three major themes (Table 2). The
206 overarching theme was that a breastfeeding-friendly city was one where breastfeeding is at the front
207 and centre of each component of the warm chain. For a city to be truly breastfeeding-friendly,
208 breastfeeding should be brought out to the forefront through prioritization and visualization of
209 breastfeeding, as well as ensuring that support for breastfeeding is continuous across the warm chain.
210 The women recognised that to achieve this, there must be clear leadership, thoughtful facilities, and a
211 welcoming culture.
212 As Dinah (Malaysia) puts it, “It is not possible for a town to be breastfeeding-friendly if it didn't come
213 from a leader.” With clear leadership, any activities related to breastfeeding will have direction and
214 be in a position of importance. She described how in her area, where she volunteers in a breastfeeding
215 support group, they will always invite a local leader to attend any event organized by the group. The
216 support demonstrated by their presence at these events may be small but sends an important message
217 towards making a city breastfeeding friendly. Priority should also be seen when considering facilities
218 for breastfeeding, where is “not on the second floor all the time” *(Olivia, Malaysia)*. A welcoming
219 culture among people in the city also brings breastfeeding to the fore front. However, this welcoming
220 culture is not always experienced by the women. There needs to be a culture change away from bottle
221 feeding if a city is to be breastfeeding friendly.
222 “Our culture is so geared to maybe more bottle feeding and not breastfeeding in public, I
223 think we need those things (stickers that say ‘Breastfeeding welcomed here”) to help it along
224 until it becomes more normalized.” *Jade, Ireland*
225 Camelia (Ireland) puts it aptly when talking about what such a breastfeeding-friendly city looks like to
226 her, “… and change the people living there!”
227 Table 2: Themes and subthemes

| Theme | Description of  theme | Subthemes |
| --- | --- | --- |
| Overarching  theme: Breastfeeding at | Overall perspective  of a city where breastfeeding is |  |

| the front and  centre of every city. | brought to the  forefront and how it can be achieved. |  |
| --- | --- | --- |
| Major Theme 1: Mothers feel supported when breastfeeding is prioritised. | Areas of support, and where they were helpful or otherwise. | ***Highest priority noted during the perinatal period:*** There was no short of support offered to healthy mothers and their newborns during this period.  ***Breastfeeding becomes secondary when the baby or mother is sick.*** |
|  |  | ***Sometimes breastfeeding support felt like an after- thought.*** This is most evident beyond the perinatal  period. |
| Major Theme 2: When breastfeeding is visible in the environment, it becomes normalized. | Normalization of breastfeeding in public. | ***Effects of breastfeeding visibility:*** Examples of how seeing other women breastfeeding made an impact on decision to breastfeed.  ***Visuals of breastfeeding as part of everyday life:*** A description of visuals of breastfeeding in media (print and electronic). |
|  |  | ***Differing views about breastfeeding in public:*** Not everyone views public breastfeeding with similar enthusiasm. |

|  |  | ***Public breastfeeding rooms are for the babies’ comfort:***  The mothers are not particularly concerned about modesty. |
| --- | --- | --- |
| Major Theme 3: There needs to be seamless breastfeeding support across the continuum of the warm chain, and all levels of  society. | Focused on the warm chain across sectors and equity of support. | ***Discontinuity of support across sectors:*** There is no continuity of support when mothers move from one sector to another.  ***Closing the gaps within the sector:*** Within each sector there are areas of support that could be addressed better.  ***Inequitable access to breastfeeding support:*** Not everyone could afford breastfeeding support. |

228

## 229 Major Theme 1: Mothers feel supported when breastfeeding is

230 **prioritised.**
231 This theme describes situations in which the mothers felt most supported during their breastfeeding
232 journey. These situations were related to the amount of priority placed on breastfeeding. The
233 subthemes are highest priority during perinatal period especially for healthy babies, breastfeeding
234 becomes secondary when the baby or mother is sick, and beyond the perinatal period, some
235 breastfeeding support felt like an after-thought.

### 236 Highest priority for breastfeeding noted during the perinatal period.

237 There was no shortage of the types of support provided for breastfeeding during the perinatal period.
238 The mothers spoke about antenatal breastfeeding classes, being given skin-to-skin immediately after
239 birth and help with breastfeeding while in the hospital after delivery. After discharge from hospital,
240 many women had a support group to go to for help to continue breastfeeding. These supports were
241 mainly directed at making sure breastfeeding gets off to a good start.
242 “I did the prenatal breastfeeding course, you know, that's offered, so that was good as a
243 starting point” *Eva, Ireland*
244 “The first golden hour they didn't take the baby away for cleaning or whatsoever, and the
245 baby got to bond with me, and she can immediately try to latch” *Melanie, Malaysia*
246 “The nurse sort of like stayed on the first time they bring her to breastfeed.…she really sat,
247 like, the whole hour (and it) was actually really helpful” *Olivia, Malaysia*
248 “My local health centre had a weekly breastfeeding group with the public health nurse, and
249 that was great” Grac*e, Ireland*
250 For those who did not have or could not access a physical in-person group, the mothers reported that
251 support from online breastfeeding groups gave them the help and encouragement to continue
252 breastfeeding.
253 “I almost quit, and I found a Facebook group for moms and I just posted there, and someone
254 told me about breastfeeding specific group and I joined that and that saved my journey”
255 *Isabelle, Ireland*
256 “Then after that, I joined [name of Facebook group]. And it was helpful because of the
257 community support, even though you don’t get it face to face.” *Elise, Malaysia*
258 Breastfeeding support groups provided not only specific support for breastfeeding but also parenting
259 and social support. In addition, it gave the mothers an opportunity to offer support to others who had
260 similar experiences.
261 “For me, [*name of group*] was a lifeline because I’ve made all of my best friends through (this
262 group)” *Nikki, Ireland*
263 “And then once you know the answer, you can also offer support (*to others*)” *Elise, Malaysia*

### 264 Breastfeeding becomes secondary when the baby or mother is sick.

265 Unlike the healthy babies, babies that required special care, breastfeeding seemed to take second place
266 in the eyes of healthcare professionals, which some mothers expressed disappointment about. The
267 mothers could understand the need to prioritize the medical problem but did not appreciate that
268 formula feeding seemed to be presented by healthcare professionals as the more appropriate way of
269 feeding. Upon reflection, when babies missed the opportunity to exclusively breastfeed, the mothers
270 expressed disappointment. It appears they wished someone gave breastfeeding a chance.
271 “So [they] would come around, they will ask you questions like, you know, indirectly they’re
272 asking you, why don't you top up with formula milk, since his reading (bilirubin) is very
273 high” *Stella, Malaysia; narrating the incidence of her baby admitted for neonatal jaundice.*
274 “But I’m kind of annoyed that (formula) was the first suggestion, instead of let’s get more
275 colostrum and syringe feed him colostrum.” *Lilia, Ireland; in response to being told that her*
276 *baby in NICU needed formula.*
277 Mothers who are unwell after delivery are sometimes separated from their newborns. In hospitals,
278 location of the wards for mothers and babies should consider breastfeeding. Although it was not
279 viewed as an inconvenience, Lilian (Ireland) described how she was “going up and down every time
280 for feed”. She was admitted post-delivery due to a medical issue and her baby required phototherapy
281 for jaundice. Another mother found it difficult to go to the hospital breastfeeding room to feed her
282 twin preterm babies.
283 “..the nursing room is quite far, maybe 2-3 minutes’ walk” *Paula, Malaysia*
284 This distance, though it may not be considered far by some, proved to be difficult for her as she was
285 post-caesarean section and had twins. She felt alone as well because her husband could not help her as
286 the room was reserved for mothers only.
287 These examples demonstrated that while there was a lot of support put in place, they were prioritized
288 for the healthy newborn and for the babies that needed hospitalization, or mothers who needed extra
289 care, breastfeeding didn’t come across as being a priority.

### 290 Sometimes breastfeeding support felt like an after-thought.

291 The experiences shared by the mothers of breastfeeding beyond the perinatal period and the hospital
292 setting highlighted the inadequacy of how some supports were delivered. In many public places,
293 breastfeeding rooms were set up for mothers to use. However, some of these rooms were so
294 inconvenient that the mothers felt that the people responsible for setting them up were merely
295 fulfilling a checklist. For example, a breastfeeding room that is locked, and the key is not easily
296 available or a room located on the top floors where it is more difficult to get to with a baby in a
297 stroller.
298 “The breastfeeding room was locked. I had to get the key from the guard but it was too slow”
299 *Ruby, Malaysia*. (She ended up breastfeeding her baby on the chair in the open)
300 “I feel like our nursing rooms should be on every floor of whatever malls or supermarkets”
301 *Olivia, Malaysia*
302 In the workplace, many women faced the similar problems and described situations where
303 breastfeeding was an afterthought. One on hand, many reported that their workplace did not stop them
304 from breastfeeding or expressing breast milk during work but on the other; however, they highlighted
305 that the support provided was not well thought through. The primary example of this was the
306 provision of breastfeeding rooms at workplaces. Unfortunately, participants reported that some
307 employers still considered the toilet to be a suitable private room to offer to their employees.
308 “Then, when I started work at six months… (my workplace) does not have a breastfeeding
309 room, but they asked me to use the staff lounge, but people came in and out.” *Violet,*
310 *Malaysia.*
311 “They asked me to pump inside the toilet and I feel that is not good for myself and the
312 breastmilk also, so I just slowly stopped” *Adeline, Malaysia*.
313 Responses to the COVID-19 pandemic provided more examples of how breastfeeding is not a priority
314 for policy makers. During the lockdown period, participants described how visiting hours at hospitals
315 were very limited and how policies included mothers of newborns admitted to the neonatal units.
316 Lactation consultants were not considered ‘essential medical services’ and there was limited access to
317 them during the pandemic. Many participants reported that even after the strict lockdown was over
318 and mothers and babies were allowed out, chairs in public places were still crossed out, and thus
319 mothers had nowhere to sit and breastfeed.
320 “I requested that I want to go to the hospital to direct-latch him, and they actually allowed me
321 to do that…of course, only during their visiting time during this COVID situation (which was
322 twice a day)” *Terri, Malaysia*.
323 “Because of pandemic restrictions, my condo (apartment) doesn't allow any visitors” *Violet,*
324 *Malaysia*.
325 “And myself I didn't experience this but in [name of city] a lot of women who were a
326 breastfeeding during the lockdown they go to the mall or something and they have crossed out
327 all the chairs, so you couldn't you didn't have spaces to sit down to breastfeed” *Isabelle,*
328 *Ireland*

## 329 Major Theme 2: When breastfeeding is visible in the

330 **environment, it becomes normalised.**
331 This theme describes how visibility of breastfeeding within a community plays a role in normalizing
332 breastfeeding, which perceived as an important measure of breastfeeding-friendliness. The mothers
333 also brought up breastfeeding in public and expressed different views about it. This theme can be
334 divided into four subthemes: effects of breastfeeding visibility, visuals of breastfeeding as part of
335 everyday life, breastfeeding in public and public breastfeeding rooms are more for the babies.

### 336 Effects of breastfeeding visibility

337 One of the main effects of breastfeeding visibility is that it creates a safe and welcoming space for
338 breastfeeding mothers. The mothers expressed this in different ways, including how seeing others
339 breastfeeding would make them not feel out of place.
340 “Just seeing people around (breastfeeding).., that's the big thing, just like that you could
341 somehow feel that no one's going to say anything to you….. kind of made you feel a bit safer”
342 *Jade, Ireland*
343 “I think that's the biggest support really would be to make it more common, to seeing
344 people…I think it has to be like that, it becomes more common and you don't feel out of
345 place” *Eva, Ireland*.
346 Another positive effect of breastfeeding visibility was the impact on one’s decision to breastfeed.
347 Having seen another person breastfeed, especially someone they know, helped some women decide to
348 breastfeed their own children, even if the encounter was many years before they had children.
349 Additionally, one mother described how when those around her saw her breastfeeding, they became
350 more positive about the behaviour.
351 “I saw my eldest sister breastfeeding and I also saw a very close friend of mine
352 breastfeeding…it was like “wah!” It was something that really imprinted in my mind that it
353 was the best thing to give to her baby” *Elise, Malaysia*
354 “My mother, I think, she didn't even like the idea of someone breastfeeding…but after me
355 being around and just feeding the baby on demand, she could see the positive aspects of
356 breastfeeding, and was very positive towards breastfeeding after that.” *Kristen, Ireland*
357 A strong visual message had a similar effect as seeing other breastfeeding women around. One mother
358 saw a poster as a teenager in a public place, and it made such an impact on her that she could recall it
359 when the time came for her to make a decision about feeding her baby.
360 “I went there for a music camp and then there was a poster with a lady feeding twins. Ever
361 since then it is my head… And I didn't think that 20 years later when I was pregnant, I
362 decided, yes, I want to breastfeed.” *Camelia. Ireland.*

### 363 Visuals of breastfeeding as part of everyday life

364 Making breastfeeding visible does not have to mean women breastfeeding in public all the time. To
365 the mothers interviewed, a big part of this was seeing the visuals of breastfeeding incorporated into
366 everyday life encounters. This includes portraying breastfeeding as the normal way of feeding a baby
367 in all forms of media. Even small gestures, like not using a bottle to indicate a baby room also helps to
368 normalize breastfeeding.
369 “It's just having things around like in books and children's books, when you see pictures of
370 breastfeeding mothers or even cartoons, dramas and soap - just where it's not made a big deal”
371 *Jade, Ireland.*
372 “Definitely advertisements on TV, I mean, we see so many advertisements on formula
373 milk…create awareness posters, billboards where it promotes breastfeeding, instead of the
374 importance of formula” S*tella, Malaysia*
375 “The baby feeding room symbol as a bottle and not a breast. It's just these kind of little things
376 that are subconscious signifiers of what is considered normal” *Amy, Ireland*

### 377 Breastfeeding in public

378 There were divided views on what breastfeeding in public meant to the mothers, in particular, whether
379 a breastfeeding room was necessary. Some believed that it is not proper to breastfeed in public, yet
380 others said they would not hesitate to do so and there were those in between, where they could
381 breastfeed without a room, but use a nursing cover or choose a discreet place. These differences
382 seemed to be related to the individual’s confidence, rather than cultural background. It is often
383 believed that Asian women were more conservative and would approach breastfeeding in public
384 differently but this was not apparent in this study. One mother from Malaysia was particularly
385 confident about breastfeeding in public and said that she would ‘stare back’ if someone was looking at
386 her breastfeeding.
387 “Breastfeeding should not be in front of everyone” *Tara, Malaysia.*
388 “And I’ve never been kind of shy or tried to hide away, because I think it's really important to
389 try and normalize breastfeeding as much as possible, I think it's encouraging when you see
390 other people out and about doing it.” *Lilian, Ireland*
391 “If someone wants to see, it’s fine, I’m just feeding my child” *Stella, Malaysia*

### 392 Public breastfeeding rooms are more for the babies.

393 Although a breastfeeding room was one of the main items on the ‘wish list’ of a breastfeeding-
394 friendly city, the reason for needing the room appeared to be more for the comfort of the baby. The
395 room, as opposed to breastfeeding in an open space, provides a quiet and comfortable environment
396 where baby can feed without distractions.
397 “(Babies) don't like to be covered up and they need to have a proper room and get seated
398 properly, then, only they can nurse well, nurse properly and all. So, baby rooms, that would
399 be better.” *Violet, Malaysia*
400 “And it's not that I’m embarrassed to feed in a restaurant. I have no problem doing that but as
401 children get older, they're more distracted.” *Grace, Ireland*
402 Where there are no rooms, mothers would seek for alternatives of this type of space. An interesting
403 alternative space was their car. It was not the most comfortable place, but several mothers described
404 how it gave them the privacy needed.
405 “So (we) try our best to comfort them and then rush to the car and latch them” *Adeline,*
406 *Malaysia, mother of twins.*
407 “Like I just did it (expressing milk) in back of my car every time that I was out and which not
408 lovely, but it's fine” *Tessa, Ireland*

## 409 Major Theme 3: Seamless and continuous breastfeeding support

410 **for everyone**
411 This theme directly addressed the warm chain of breastfeeding. Seamless breastfeeding support is
412 essential throughout the continuum of care and all level of society. The women highlighted gaps in
413 support, and these were viewed negatively. These gaps can be seen in 3 main areas, corresponding to
414 the subthemes: continuity of support across sectors, closing the gaps within the sector, and equity of
415 access to breastfeeding support. Sectors refer to the three main sectors in the warm chain (Fig 1).

### 416 Discontinuity of support across sectors

417 One of the obvious issues identified from the interviews was that breastfeeding supports are often in-
418 silo, not taking account the fluidity of movement of the breastfeeding dyad across the continuum of
419 the warm chain. For example, the mothers noted that the positive support they received while at the
420 hospital came to an abrupt stop once the new mother and baby went home. The transition from
421 hospital to home is not easy for all women and for some, the connection to the community support
422 group was not readily known to them and some weren’t provided information about breastfeeding
423 support groups in the community while they were in the hospital. Similarly, transitioning from home
424 to work was described as challenging and they could not always find the support they needed. Again,
425 this highlights that having a seamless support system helping mothers to transition from one sector to
426 another is important.
427 “So, once I went home, we just figure it out yourself but while they were there (i.e. at the
428 hospital), they were helpful” *Camelia, Ireland.*
429 “I think it would be a nice thing if the people from those groups could drop by their hospital
430 once in a while to meet the people who are there” *Isabelle, Ireland.*
431 “I find there was very little support for extended breastfeeding. I went back to (support group)
432 breastfeeding meeting at around nine months before I went back to work, looking for advice,
433 but everybody there had tiny babies and nobody who was still breastfeeding was still
434 attending the classes.” *Kristen, Ireland*

### 435 Closing the gaps within the sector

436 Within each sector, there were gaps in the quality and consistency of the support provided for the
437 mothers. In the community, while there were support groups and access to lactation consultants, there
438 was difficulty in accessing donor breastmilk. A mother from who could not find donor breast milk for
439 her newborn described how there are “blood banks out there, but there are no breast milk banks.”
440 In the healthcare sector, where breastfeeding support was reported as most constant, the quality of
441 support given was inconsistent. For example, preterm babies were encouraged to be given expressed
442 breast milk, but the mother was not taught or assessed if she knew how to express her milk. Health
443 professionals, particularly general practitioners, lacked the expertise to detect problems related to
444 breastfeeding, much to the disappointment of the mothers.
445 “They just passed me a syringe and then close the curtain. I just hide inside and hand express
446 myself” *Adeline, Malaysia.*
447 “I saw four GPs about it, and none of them diagnosed it as a milk allergy…I just felt, that I
448 was let down a little bit even in terms of being taken seriously about the concerns” *Amy,*
449 *Ireland*
450 In the workplace, policy and practice seemed to be incongruent. At the time of the interviews,
451 breastfeeding breaks were available nationally in Ireland until the baby was 26 weeks old. There were
452 some sectors (e.g. teachers, civil servants, and healthcare workers), who had worked through their
453 unions to have breastfeeding breaks until their child was 104 weeks (33). However, utilization of
454 these breaks can be challenging as described by the teachers in this study.
455 “A lot of secondary school teachers aren't taking up the breastfeeding breaks because it's too
456 much of an imposition on their colleagues, and on their students” *Lilian, Ireland*
457 “So there wouldn't be anyone to supervise my class. I could have arranged and management
458 were really supportive, but I just felt it wasn't worth my while to do that, this time, and also
459 because my daughter was a bit older (over 1 year), I felt she was fine without it just for those
460 few hours” *Grace, Ireland*

### 461 Inequitable access to breastfeeding support

462 Equity in access to skilled lactation support was described as an issue by some of the mothers. In
463 Ireland, there were few lactation consultants in the public health system and many women would
464 engage private lactation consultation services. In Malaysia, all lactation consultations are privately
465 based. This means that many mothers had to pay for access to the lactation consultants. The extra cost
466 would mean that some women were not able to afford this service.
467 “Yeah, it was just that was for me breastfeeding was really handy but I couldn't afford a
468 lactation consultant because that's 50 euro - that goes towards food.” *Nikki, Ireland*
469 Inequity in breastfeeding support was also apparent for mothers in the informal work sector. As
470 described earlier, there was strong policy for workplace support in Ireland. In Malaysia, there is a no
471 clear policy for breastfeeding other than mandatory maternity leave, but many formal workplaces
472 would provide minimal support at the workplace such as a dedicated room and freedom to express
473 breast milk at work. However, for women in the informal workforce, these are not present and success
474 or failure to continue breastfeeding depended on the individual mother’s determination and resilience.
475 For example, Ruby from Malaysia works on-the-go, sometimes between 7am until 12 midnight. She
476 would express breast milk in her car or at shopping malls while having her lunch. She described
477 keeping her expressed breast milk in a cooler box in her car, occasionally having to add ice into the
478 box midway through her day. In contrast, Tara also from Malaysia, who runs a small food business by
479 the road does have any way of expressing breast milk while she is at work.
480 “I breastfeed him when I am back home but when I am out (working), he drinks formula milk
481 from the bottle at home” *Tara, Malaysia.*

# 482 Discussion:

483 The ideal breastfeeding-friendly city was epitomized in the themes developed. Although the
484 participants in our study came from two very different cultures, they generally wished that
485 breastfeeding be prioritized and normalized and continuously supported in the place where they live.
486 One of the main characteristics of a breastfeeding friendly city described by mothers was a place to
487 breastfeed their babies freely. While many would have preferred that breastfeeding could be
488 normalized and thus no need to shy away from breastfeeding in public, this was idealistic rather than
489 practical. Therefore, they had a pragmatic request for breastfeeding rooms to be available around the
490 city. However, the women in our study had another reason for wanting a private room which is related
491 to the baby. This is phenomenon is not reported in other studies about breastfeeding in public (34). It
492 is important to note that the general acceptance of public breastfeeding in Ireland has been reported as
493 good (35), while in Malaysia, women have been reported to generally feel uncomfortable about
494 breastfeeding in public (36, 37). The experiences shared, and views expressed by the women in our
495 study about breastfeeding while out and about resonated with other studies. Sheehan *et al*. explored
496 the views of first-time expectant mothers and their families’ views on breastfeeding in public (38).
497 This Australian study found that not everyone can accept breastfeeding in public and there’s a general
498 view that some discretion is needed. In that study, the car was also named as one such space to
499 breastfeed away from the public view. Considering the point of view of the public, the acceptance of
500 public breastfeeding is variable. A study by Morris *et al* found that most netizens in the United
501 Kingdom do not immediately welcome this (39). Therefore, creating accessible and welcoming public
502 spaces for women to breastfeed is needed in a breastfeeding-friendly city, whether this includes a
503 public breastfeeding room or not.
504 It is clear from epidemiological evidence that where there is high priority for and concentration of
505 breastfeeding support, there will be higher breastfeeding rates. For example, the breastfeeding
506 initiation rate before hospital discharge (at birth and within one day of birth) in Malaysia was 89.6%
507 in 2016, but by six months, the prevalence of exclusive breastfeeding dropped to 47.1% (28). Similar
508 trend is seen in Ireland, with 63.1% of babies breastfed at birth in 2021, but by three months, 31.1%
509 were exclusively breastfed (27). Evidence from other studies looking at intention to breastfeed and
510 actual breastfeeding duration also demonstrated that a majority of the mothers started well but stopped
511 breastfeeding early (6). Hence, the importance of having sustained and continuous support for
512 breastfeeding beyond the perinatal period. Having a city that support breastfeeding across all time
513 points in the breastfeeding journey could improve these outcomes.
514 The question arises if cities are currently built to support breastfeeding. An ethnographic study of a
515 city in Wales, United Kingdom found that the city centre and its public transport areas were not the
516 most ‘friendly’ places for women to breastfeed (40). The study reported a busy and small waiting
517 room at the railway station with chairs facing each other, making it difficult for a mother to
518 “discreetly” breastfeed. It is interesting to note that the researcher found that while cafés are
519 comfortable and private enough to breastfeed, the rapid pace of change of customers in a particular
520 café could potentially mean that mothers sitting down to breastfeed their babies for up to twenty
521 minutes may be frowned upon by the staff. Therefore, providing the infrastructure alone would not be
522 enough and a culture change of accepting that breastfeeding is what babies do normally is also
523 needed.
524 Many of the women in our study suggested that one way to normalize breastfeeding is to have
525 pictures of breastfeeding as posters in public spaces or incorporated into everyday life such as books
526 and television. An analysis of images of breastfeeding by Giles offers a good insight into what cities
527 can do when using pictures and images of breastfeeding (41). A picture tells a thousand words;
528 therefore, the choice of image portraying breastfeeding is important. Many images show breastfeeding
529 women alone, with the absence of other people, especially non-breastfeeding individuals. According
530 to Giles, this portrays breastfeeding as something private, even though the pictures are publicly
531 displayed. To change culture on breastfeeding, images of breastfeeding women engaging socially
532 with non-breastfeeding community members could be the way forward. Some examples would be the
533 works of a Malaysian artist, Chuah Thean Teng, who often depicts a breastfeeding mother in daily life
534 activities (42), as well as Irish artist Fiona Carey with her collection on “Everyday Breastfeeding”
535 (https://[www.fionacarey.com/illustration).](http://www.fionacarey.com/illustration))
536 A supportive and enabling environment is essential for breastfeeding success, no matter the
537 geographic location. However, the specific needs may differ from place to place depending on the
538 social, economic and cultural setting. This study exemplified the difference between a high-income
539 and middle-income country from two different continents. In the context of workplace support, there
540 was a clear difference. Women in Ireland were privileged to a much longer maternity leave (at least
541 six months) and breastfeeding breaks. In contrast, Malaysian women had only two months maternity
542 leave (note: a new legislation after the interview provides 90 days of paid maternity leave) and no
543 breastfeeding breaks. Data from the International Labour Office showed that in 2022, 67.8% of Irish
544 women received maternity benefits compared to 46.5% of Malaysian women (data from 2020) (43).
545 Another difference is the education levels of the women from both countries. There was a
546 significantly higher number of women with a higher degree in Ireland compared to Malaysia. This is
547 not likely to have occurred by chance because Ireland ranks as one of the top 10 countries with the
548 highest number of PhD holders (44). The difference in education level could have contributed to how
549 much the women ‘tolerated’ any lack of support and what they did to overcome that. Women who are
550 not cognisant of what they could have received in terms of support would generally accept what was
551 available and would not seek out further support. From our sample, women with higher education or
552 those who had more prior knowledge of breastfeeding support seemed to seek out for themselves
553 support that was missing.
554 Although the specific examples highlighted the differences, the main themes remain similar regardless
555 of the location. Even with the obvious socioeconomic and cultural differences between the two
556 countries, breastfeeding women had similar perspectives when it came to the types of support they
557 require. Other international studies such as our study also demonstrated that the support needs of
558 breastfeeding women are universal (45, 46).
559 A key observation from the interviews were that almost all of the women demonstrated an
560 extraordinary determination to breastfeed, despite facing obstacles and challenges. They went to great
561 lengths to make breastfeeding work for them. Some even had to ‘fight’ for their right to breastfeed,
562 when told by healthcare professionals that they should introduce formula. The strong determination
563 fits the description of the ‘passionate’ group of women as reported by another study in Malaysia (47).
564 However, not all breastfeeding women will fall into the ‘passionate’ group. Success in breastfeeding
565 should not be dependent on individual motivation. The original breastfeeding friendly statement, the
566 Innocenti Declaration, called for efforts to increase the individual mother’s confidence and stated that
567 “obstacles to breastfeeding within the health system, the workplace and the community must be
568 eliminated” (48). From the interviews in this study, there were many instances of obstacles to
569 breastfeeding still present even though it has been over 30 years since the declaration. An enabling
570 environment would negate the need for the individual mother to be incredibly passionate or motivated
571 to succeed in breastfeeding.
572 At the time of the interviews, restrictions related to Covid 19 were still present. Many of the mothers
573 had their babies during the peak of the pandemic. The experiences of the women during that time
574 demonstrated how an emergency situation amplified existing inadequacies of breastfeeding support.
575 At that time, breastfeeding was not considered a priority or essential healthcare. Incidences shared by
576 some women such as restricted visiting hours, and not allowing lactation consultants to enter
577 apartment building because it was considered non-essential, were some examples. Part of the issue
578 was the slow understanding of the impact of the virus on breastfeeding (49). By the time policies
579 changed, many babies would have missed the opportunity to breastfeed, some exclusively and some
580 altogether. A cross-sectional survey of post-partum women in 5 countries found that they experienced
581 poor breastfeeding support during the pandemic (50). Breastfeeding during other emergencies such as
582 natural disasters and war also faced similar challenges (51, 52). One issue observed from these
583 situations is the uncontrolled distribution of breastmilk substitutes taking precedence over helping
584 mothers to overcome difficulties in breastfeeding (53). This again highlights the fact that
585 breastfeeding needs to be at the front and centre where mothers and babies are concerned.

## 586 Strengths and Limitations

587 The methods used in this study accounted for potential cultural differences between the two countries.
588 Our cross-cultural research team had either worked or are currently working in one of the countries.
589 We also ensured that the interviews would be comprehensible to all participants by piloting the
590 interviews with women from both countries. Consistency was maintained by having a single
591 researcher conduct all interviews. The themes were developed specifically by two authors, one each
592 from Ireland and Malaysia. In presenting the results, additional background information and the
593 contextual nuances of the quotes were added for a deeper understanding of our findings.
594 The primary limitation of this study is that we interviewed only women who are currently
595 breastfeeding or had breastfed before. This may present a biased view as they were more likely to be
596 those who are highly motivated to breastfeed. However, among them, they had their fair share of
597 negative experiences. The unsuccessful experiences with breastfeeding gave them insights into the
598 type of breastfeeding support needed.

# 599 Conclusions:

600 The views and experiences of breastfeeding women across two continents demonstrated the
601 importance of prioritized, and continuous support throughout the breastfeeding journey. Their hopes
602 and aspirations of a breastfeeding-friendly city would be useful for cities to consider when developing
603 or implementing breastfeeding support programmes. The findings can also be used to guide
604 development of indicators of a breastfeeding-friendly city.

# 605 Acknowledgements:

606 Ting Kai Xin for translation of the Malay interviews.
607 Page administrators of the two Facebook groups we used to recruit the participants.
608 All the women who responded to our call for participants and those who took time for the interviews.

609

# 610 References:

611 1.	Victora CG, Bahl R, Barros AJD, França GVA, Horton S, Krasevec J, et al. Breastfeeding in
612 the 21st century: epidemiology, mechanisms, and lifelong effect. The Lancet. 2016;387(10017):475-
613 90.
614 2.	O'Reilly SL, McNestry C, McGuinness D, Killeen SL, Mehegan J, Coughlan B, et al.
615 Multicomponent perinatal breastfeeding support in women with BMI >25: The Latch On multi-centre
616 randomised trial. Bjog. 2024.
617 3.	Yelverton CA, Killeen SL, Feehily C, Moore RL, Callaghan SL, Geraghty AA, et al.
618 Maternal breastfeeding is associated with offspring microbiome diversity; a secondary analysis of the
619 MicrobeMom randomized control trial. Front Microbiol. 2023;14:1154114.
620 4.	Walters DD, Phan LTH, Mathisen R. The cost of not breastfeeding: global results from a new
621 tool. Health Policy and Planning. 2019;34(6):407-17.
622 5.	World Health Organization, Unicef. Global strategy for infant and young child feeding.
623 whqlibdocwhoint/publications/2003/9241562218pdf (accessed 2014)2003.
624 6.	Kay MC, Cholera R, Flower KB, Yin HS, Rothman RL, Sanders LM, et al. Are Low-Income,
625 Diverse Mothers Able to Meet Breastfeeding Intentions After 2 Months of Breastfeeding?
626 Breastfeeding medicine : the official journal of the Academy of Breastfeeding Medicine.
627 2020;15(7):435-42.
628 7.	Shohaimi NM, Mazelan M, Ramanathan K, Meor Hazizi MS, Leong YN, Cheong XB, et al.
629 Intention and practice on breastfeeding among pregnant mothers in Malaysia and factors associated
630 with practice of exclusive breastfeeding: A cohort study. PloS one. 2022;17(1):e0262401.
631 8.	Hemmingway A, Fisher D, Berkery T, Dempsey E, Murray DM, Kiely ME. A detailed
632 exploration of early infant milk feeding in a prospective birth cohort study in Ireland: combination
633 feeding of breast milk and infant formula and early breast-feeding cessation. British Journal of
634 Nutrition. 2020;124(4):440-9.
635 9.	Tan ML, Foong SC, Foong WC, Ho JJ. Use of Galactagogues in a Multi-Ethnic Community
636 in Southeast Asia: A Descriptive Study. International journal of women's health. 2022;14:1395-404.
637 10.	Tan KL. Factors associated with exclusive breastfeeding among infants under six months of
638 age in peninsular malaysia. Int Breastfeed J. 2011;6(1):2.
639 11.	Perrine CG, Scanlon KS, Li R, Odom E, Grummer-Strawn LM. Baby-Friendly Hospital
640 Practices and Meeting Exclusive Breastfeeding Intention. Pediatrics. 2012;130(1):54-60.
641 12.	McGovern L, Geraghty A, McAuliffe F, O'Reilly S. An exploration of prenatal breastfeeding
642 self-efficacy: a scoping review protocol. Open Res Eur. 2022;2:91.
643 13.	O'Sullivan EJ, Alberdi G, Scully H, Kelly N, Kincaid R, Murtagh R, et al. Antenatal
644 breastfeeding self-efficacy and breastfeeding outcomes among mothers participating in a feasibility
645 breastfeeding-support intervention. Ir J Med Sci. 2019;188(2):569-78.
646 14.	Bartels HC, Kennelly MA, Killeen SL, Lindsay KL, Crowley RK, McAuliffe FM. An
647 mHealth-Supported antenatal lifestyle intervention may be associated with improved maternal sleep in
648 pregnancy: Secondary analysis from the PEARS trial. Bjog. 2022;129(13):2195-202.
649 15.	A warm chain for breastfeeding. The Lancet. 1994;344(8932):1239-41.
650 16.	Hector D, King L, Webb K, Heywood P. Factors affecting breastfeeding practices. Applying
651 a conceptual framework. NSW Public Health Bulletin. 2005;16(4):52-5.
652 17.	Rollins NC, Bhandari N, Hajeebhoy N, Horton S, Lutter CK, Martines JC, et al. Why invest,
653 and what it will take to improve breastfeeding practices? The Lancet. 2016;387(10017):491-504.
654 18.	Pérez-Escamilla R, Curry L, Minhas D, Taylor L, Bradley E. Scaling up of breastfeeding
655 promotion programs in low- and middle-income countries: the "breastfeeding gear" model. Advances
656 in nutrition (Bethesda, Md). 2012;3(6):790-800.
657 19.	Al Ghazal H, Rashid S, Ruf E. The Sharjah Baby-Friendly Campaign: A Community-Based
658 Model for Breastfeeding Promotion, Protection, and Support. Breastfeeding medicine : the official
659 journal of the Academy of Breastfeeding Medicine. 2015;10(9):437-41.
660 20.	Knežević MŠ, Jeleč V, Knežević MŠ, Armano G, Bošnjak AP, Rončević I. 44 Breastfeeding
661 friendly City of Zagreb. Archives of Disease in Childhood. 2021;106(Suppl 2):A19-A.
662 21.	Roberts L. Boobylicious thumbs-up. Northern Territory News (Australia). 2016 9 July;Sect.
663 News.
664 22.	Tan ML, Mohd Shukri IA, Ho JJ, O'Sullivan EJ, Omer-Salim A, McAuliffe Fionnuala M.
665 What makes a city ‘breastfeeding-friendly’? A scoping review of indicators of a breastfeeding-
666 friendly city. Maternal & child nutrition. 2023;n/a(n/a):e13608.
667 23.	UNICEF. The Baby Friendly Hospital Initiative 1991 [Available from:
668 https://[www.unicef.org/programme/breastfeeding/baby.htm#10.](http://www.unicef.org/programme/breastfeeding/baby.htm#10)
669 24.	Price A, Clarke M, Staniszewska S, Chu L, Tembo D, Kirkpatrick M, et al. Patient and Public
670 Involvement in research: A journey to co-production. Patient Educ Couns. 2022;105(4):1041-7.
671 25.	Tan ML, Foong SC, Foong WC, Tay GY, Ho JJ, Omer-Salim A, et al. Identifying indicators
672 of a breastfeeding-friendly city: A Delphi survey, 8th ABM/EABM European Regional Conference
673 May 11–13, 2023 Split, Croatia. Breastfeeding Medicine. 2023;18(5):A-1-A-25.
674 26.	Organization WH. Indicators for assessing infant and young child feeding practices (Part 1
675 Definitions). Washington D.C.: World Health Organization; 2008.
676 27.	WBTi Ireland Steering and Core Group. WBTi Assessment Report Ireland. Ireland 2023.
677 28.	Institute for Public Health NIoH, Ministry of Health, Malaysia. National Health and
678 Morbidity Survey (NHMS) 2016: Maternal and Child Health. Vol II: Findings. 2016. p. 272.
679 29.	Baker JL, Michaelsen KF, Sørensen TIA, Rasmussen KM. High prepregnant body mass index
680 is associated with early termination of full and any breastfeeding in Danish women2. The American
681 Journal of Clinical Nutrition. 2007;86(2):404-11.
682 30.	Turcksin R, Bel S, Galjaard S, Devlieger R. Maternal obesity and breastfeeding intention,
683 initiation, intensity and duration: a systematic review. Maternal & child nutrition. 2014;10(2):166-83.
684 31.	Lumivero. NVivo. 13 ed2020.
685 32.	Braun V, Clarke V. Using thematic analysis in psychology. Qualitative Research in
686 Psychology. 2006;3(2):77-101.
687 33.	Work Life Balance and Miscellaneous Provisions Act 2023, (2023).
688 34.	Dykes C, Ny P, Hauck YL, Kuliukas L, Gallagher L, Brady V, et al. Women’s perceptions of
689 factors needed to encourage a culture of public breastfeeding: a cross-sectional study in Sweden,
690 Ireland and Australia. International Breastfeeding Journal. 2023;18(1):49.
691 35.	O'Sullivan EJ, O'Grady A, Pawlak K, Kearney JM. A Qualitative Exploration of the Attitudes
692 and Experiences of Polish Breastfeeding Mothers in Ireland. Journal of human lactation : official
693 journal of International Lactation Consultant Association. 2021;37(2):370-9.
694 36.	Ishak S, Adzan NA, Quan LK, Shafie MH, Rani NA, Ramli KG. Knowledge and beliefs
695 about breastfeeding are not determinants for successful breastfeeding. Breastfeeding medicine : the
696 official journal of the Academy of Breastfeeding Medicine. 2014;9:308-12.
697 37.	Tan KL, Jr. Factors Associated with Non-exclusive Breastfeeding among 4-Week Post-
698 partum Mothers in Klang District, Peninsular Malaysia. Malaysian journal of nutrition.
699 2009;15(1):11-8.
700 38.	Sheehan A, Gribble K, Schmied V. It’s okay to breastfeed in public but…. International
701 Breastfeeding Journal. 2019;14(1):24.
702 39.	Morris C, Zaraté de la Fuente GA, Williams CET, Hirst C. UK Views toward Breastfeeding
703 in Public:An Analysis of the Public’s Response to the Claridge’s Incident. Journal of Human
704 Lactation. 2016;32(3):472-80.
705 40.	Grant A. Breasts and the city: an urban ethnography of infant feeding in public spaces within
706 Cardiff, United Kingdom. International breastfeeding journal. 2021;16(1):37.
707 41.	Giles F. Images of women breastfeeding in public: solitude and sociality in recent
708 photographic portraiture. International Breastfeeding Journal. 2018;13(1):52.
709 42.	Wenyan G, Wan Samiati Andriana WMD, Tahir A. The Spirit and Elements of Malaysian
710 Multiculturalism in Chuah Thean Teng’s Batik Paintings. Ideology Journal. 2023;8(2):191-202.
711 43.	International Labour Organization. ILOSTAT Geneva: United Nations; 2024 [Available
712 from: https://ilostat.ilo.org/data/country-profiles/.
713 44.	OECD. Education at a Glance 20192019.
714 45.	Hauck YL, Blixt I, Hildingsson I, Gallagher L, Rubertsson C, Thomson B, et al. Australian,
715 Irish and Swedish women’s perceptions of what assisted them to breastfeed for six months:
716 exploratory design using critical incident technique. BMC public health. 2016;16(1):1067.
717 46.	Lawlor N, Prihodova L, Byrne D, Etherton M, Rahill F, Wilson C, et al. A qualitative analysis
718 of women’s postnatal experiences of breastfeeding supports during the perinatal period in Ireland.
719 PloS one. 2023;18(7):e0288230.
720 47.	Sulaiman Z, Liamputtong P, Amir LH. Timing of return to work and women's breastfeeding
721 practices in urban Malaysia: A qualitative study. Health & Social Care in the Community.
722 2018;26(1):48-55.
723 48.	United Nations Children's Fund. 1990-2005 Celebrating the Innocenti Declaration on the
724 Protection, Promotion and Support of Breastfeeding: Past achievements, present challenges and
725 priority actions for infant and young child feeding 2nd ed. Geneva: Innocenti Publications; 2007.
726 49.	Klingenberg C, Tembulkar SK, Lavizzari A, Roehr CC, Ehret DEY, Vain NE, et al. COVID-
727 19 preparedness-a survey among neonatal care providers in low- and middle-income countries.
728 Journal of perinatology : official journal of the California Perinatal Association. 2021;41(5):988-97.
729 50.	Coca KP, Lee EY, Chien LY, Souza ACP, Kittikul P, Hong SA, et al. Postnatal women's
730 breastfeeding beliefs, practices, and support during the COVID-19 pandemic: a cross-sectional
731 comparative study across five countries. Int Breastfeed J. 2022;17(1):58.
732 51.	Rabbani A, Padhani ZA, A Siddiqui F, Das JK, Bhutta Z. Systematic review of infant and
733 young child feeding practices in conflict areas: What the evidence advocates. BMJ open. 2020;10(9).
734 52.	Hwang CH, Iellamo A, Ververs M. Barriers and challenges of infant feeding in disasters in
735 middle- and high-income countries. Int Breastfeed J. 2021;16(1):62.
736 53.	Sulaiman Z, Mohamad N, Ismail TA, Johari N, Hussain NH. Infant feeding concerns in times
737 of natural disaster: lessons learned from the 2014 flood in Kelantan, Malaysia. Asia Pacific journal of
738 clinical nutrition. 2016;25(3):625-30.

739

Figure 1	[Click here to access/download;Figure;Figure-1.tiff](https://www2.cloud.editorialmanager.com/pone/download.aspx?id=36536046&guid=ce038f0c-294c-470b-8ca0-0fd4b3fd43df&scheme=1)[<https://www2.cloud.editorialmanager.com/pone/download.aspx?id=36536046&guid=ce038f0c-294c-470b-8ca0-0fd4b3fd43df&scheme=1>](https://www2.cloud.editorialmanager.com/pone/download.aspx?id=36536046&guid=ce038f0c-294c-470b-8ca0-0fd4b3fd43df&scheme=1)
